# Silk‐Enabled Conformal Multifunctional Bioelectronics for Investigation of Spatiotemporal Epileptiform Activities and Multimodal Neural Encoding/Decoding

**DOI:** 10.1002/advs.201801617

**Published:** 2019-03-07

**Authors:** Zhifeng Shi, Faming Zheng, Zhitao Zhou, Meng Li, Zhen Fan, Huanpeng Ye, Shan Zhang, Ting Xiao, Liang Chen, Tiger H. Tao, Yun‐Lu Sun, Ying Mao

**Affiliations:** ^1^ Department of Neurosurgery Huashan Hospital of Fudan University Wulumuqi Zhong Road 12 Shanghai 200040 China; ^2^ State Key Laboratory of Transducer Technology Shanghai Institute of Microsystem and Information Technology Chinese Academy of Sciences Shanghai 200050 China; ^3^ The Rowland Institute Harvard University Cambridge MA 02142 USA; ^4^ State Key Laboratory of Mechanical System and Vibration Shanghai Jiao Tong University Shanghai 200240 China; ^5^ School of Graduate Study University of Chinese Academy of Sciences Beijing 100049 China; ^6^ The Key Laboratory of Resource Chemistry of Ministry of Education Shanghai Normal University Shanghai 200234 China; ^7^ Center of Materials Science and Optoelectronics Engineering University of Chinese Academy of Sciences Beijing 100049 China; ^8^ School of Physical Science and Technology Shanghai Tech University Shanghai 200031 China; ^9^ State Key Laboratory of Integrated Optoelectronics College of Electronic Science and Engineering Jilin University 2699 Qianjin Street Changchun 130012 China

**Keywords:** brain–machine interfaces, conformal transient bioelectronics, electrocorticogram, neural decoding, silk

## Abstract

Flexible electronics can serve as powerful tools for biomedical diagnosis and therapies of neurological disorders, particularly for application cases with brain–machine interfaces (BMIs). Existing conformal soft bioelectrodes are applicable for basic electrocorticogram (ECoG) collecting/monitoring. Nevertheless, as an emerging and promising approach, further multidisciplinary efforts are still demanded for in‐depth exploitations with these conformal soft electronics toward their practical neurophysiological applications in both scientific research and real‐world clinical operation. Here, clinically‐friendly silk‐supported/delivered soft bioelectronics are developed, and multiple functions and features valuable for customizable intracranial applications (e.g., biocompatible and spontaneously conformal coupling with cortical surface, spatiotemporal ECoG detecting/monitoring, electro‐neurophysiological neural stimulating/decoding, controllable loading/delivery of therapeutic molecules, and parallel optical readouts of operating states) are integrated.

Advances in soft and/or flexible electronics enable powerful tools to promote developments of neurophysiology and relevant biomedical diagnosis and therapies of neurological disorders (e.g., epilepsy, Alzheimer's disease, Parkinson's disease, depression, and peripheral nervous disorders), particularly for application cases with brain–machine interfaces (BMIs).^1^ Neural tissues or organs like brains, along with many other biological tissues, are of soft mechanics generally with kPa level modulus and convoluted topography, which bring about their large mismatch and challenging integration with the traditionally hard electronics.^2^ Well‐working BMIs require interfacing electronic devices to be soft for their intimate—and preferably noninvasive—wrapping onto these complex biosurfaces.^3^ The emerging spontaneously conformal strategies via silk significantly facilitate to solve this challenging issue by reconciling inorganic electronic devices (usually hard but better electroperforming^4^) with cortical surfaces.^5^ Compared with conventional clinical subdural electrode arrays for BMIs (≈1 cm spacings and ≈0.35 cm diameters),^6^ these conformal microelectrodes own microscale configuration and detecting spatial resolution, compliant soft features, and biocompatible material designs.^7^, ^8^ So, they provide compatible and stable in vivo operations directly on brain tissue, improved nonpenetrating/invasive coupling with irregularly curvilinear cortical surfaces, and importantly, high BMI signal quality.^9^

The transient supporting and controllable delivery can be enabled well with plain silk films for implantable electronic and/or optical devices.^10^, ^11^ They are two important utilizations among the diverse functions and characteristics via silk's special features (e.g., facile micro/nanostructuring, programmable degradation, and biospecimen loading/stabilizing/releasing).^11^, ^12^ While silk‐based transient supporting/delivery has been well investigated, the capability of integratively platforming multiple functions is still less explored. The function integration of bioelectronics is highly useful for applications in relatively sensitive, space‐limited, and complex physiological environments, for instance, intracranial electrocorticogram (ECoG) BMIs. Existing conformal soft bioelectrodes are applicable for basic ECoG collecting/monitoring.^5^ Nevertheless, as an emerging and promising approach, further multidisciplinary efforts are still demanded in‐depth exploitations with these conformal soft electronics toward their practical neurophysiological applications in both scientific research and clinical operation. For example, the implementation of neural/brain decoding highly relies on bioelectrical‐detecting performances of BMIs (e.g., detecting fidelity and signal‐to‐noise ratio (SNR), in vivo compatibility, and stability with low collateral impact or damage).^3^, ^13^ Although potentials are exhibited,^14^ it is still less exploited with these conformal electronic devices. Meanwhile, besides functions associated with bioelectrical detection, an important aspect of BMIs' applications is to introduce artificial stimulations to neural cells, tissues, and organs.^15^ Moreover, multifunction integration is considerably in favor of multipurpose operations (e.g., monitoring, stimulation, and in situ treatments) within only one device or system in the space‐limited and complicated biological, especially intracranial, environments.^16^

We herein develop the clinical‐friendly silk‐supported/delivered soft bioelectronics, and integrate multiple functions and features valuable for customizable intracranial applications (e.g., biocompatible and spontaneously conformal coupling with cortical surface, spatiotemporal ECoG detecting/monitoring, electro‐neurophysiological neural stimulating/decoding, controllable loading/delivery of therapeutic molecules, and parallel optical readouts of operating states). Compared with previously reported work,^5^, ^17^, ^18^ silk not only functions for originally supporting and conformally delivering these thin metallic devices, but also serves as a vehicle for controllable in situ drug delivery, a monitor (by fabricating with diffractive optical element patterns) for electrodes' conformal attachments and drug release, which exhibits the “all‐in‐one” capability of silk materials. Based on their well‐tailored ECoG capability and the principal component analysis (PCA) of detected neural‐activity information, neural decoding is operated to dynamically reveal brain activities of the rat under various states and/or stimulations (e.g., an idle state, visual and electrical stimulations, and ketamine anesthetizations with different doses).

In the experiments, thin layers of brain electronics (for detection and stimulation) are lithographically fabricated with the device configuration as shown in **Figure** **1**a (see more details in the “Experimental Section” and Figures S2–S4 in the Supporting Information). A typical as‐fabricated device is composed of ≈30 µm thick silk substrate, Au/Cr (150 nm/15 nm) stimulating/detecting electrode layers, and 2 µm thick polyimide (PI) supporting/isolating layers. The ultrathin PI interlayers, with the mesh layouts conformal with electrodes, provide their superior compliant mounting onto the cortex surface. But practically, their extreme mechanical softness and fragility make it difficult to be manipulated for processing, connecting, or in vivo implanting. The silk substrate is biocompatible and resorbable in a physiological environment, mechanically supporting and subsequently delivering these brain electronics noninvasively and conformally onto cortex. Therapeutic molecules (e.g., phenobarbital here) can be loaded as needed in silk substrate and/or packages for localized programmable releasing. Compared with other biodegradable hydrogels or polymers, silk has its special or even unique merits for BMIs' applications. First, silk has high but controllable mechanical strength even when it is prepared to be ultrathin (≈4 µm).^19^ Second, unlike a lot of synthetic or semisynthetic hydrogels or polymers (e.g., polylactic acid) producing H_2_O (which may induce localized hydrops and edema) after in vivo degradation,^20^, ^21^ the products after silk's degradation are amino acids,^22^ which can be totally absorbable in vivo. Third, silk materials have been proved to be well compatible to diverse processing and advanced fabricating technologies,^12^, ^23^, ^24^ which help greatly to integrate functions toward silk‐based “all‐in‐one” multifunctional bioelectronics including, but not limited to, BMIs. Moreover, a lot of therapeutic drugs can be loaded in silk matrix and then controllably released.^25^, ^26^

**Figure 1:**
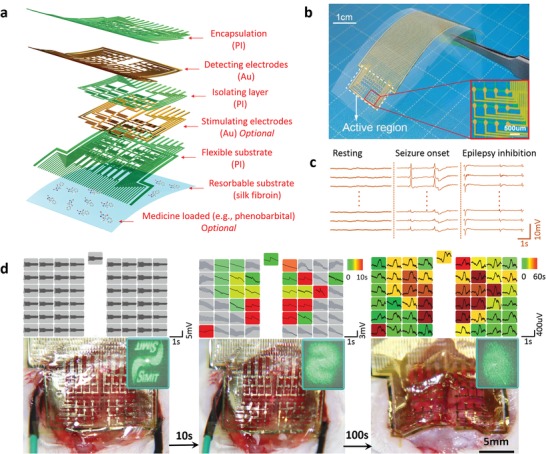
Schematic of device configuration and conformal mounting/working on rat brain. a) An explosive view of device configuration. b) Single‐layer brain electrode meshes on silk film; inset, the array of Au electrical probes. c) ECoG signals collected under resting, epileptic seizure onset, and epilepsy inhibition statuses. d) Device images and ECoG signals during conformal mounting progress on rat brain; left, middle, and right columns, respectively, show statuses before, during (promoted by adding normal saline), and after compliant mounting. Colors of signal lattices are associated with the time experienced to provide effective ECoG signals (gray, noncontact).

Diverse electronic devices are facilely customized toward integration of bioelectrical functions, for example, multiple electrode layers for stimulating nervous impulses and/or detecting ECoG signals. Therefore, the silk‐supported/delivered conformal brain‐integrated electronics work as a promising strategy for multifunctionlization (e.g., neural stimulation, ECoG detection, and drug delivery). The PI layer on the top surface of the silk matrix and the isolating one between Au/Cr layers are customized to be ≈2 µm thick. This configuration provides sufficient mechanical support after delivery. Meanwhile, its purposely soft feature enables the spontaneously compliant wrapping and contact on complex cortex surface, as well as superior mechanical biocompatibility within soft brain tissues.

Figure 1b shows an example of single‐layer Au/Cr 49‐channel electrode mesh before delivery. It is constructed eudipleurally in an ≈1 × 1 cm^2^ area for in situ ECoG detection on the rat brain. In the dry state, the silk substrate sufficiently supports the thin electronics fabricated on top to keep flat and properly hard before using, facilitating the in vivo and in situ operations. To collect and map ECoG signals in/across two brain hemispheres, the left or right part of the meshes has 24 channels each and one probing channel is positioned in the middle aiming at the joint region. The electrode arrays are placed directly on the exposed brain of a rat after craniotomy, and then silk is dissolved with saline for conformally implanting (detailed description in “Experimental Section” and Figures S5 and S11 in the Supporting Information). With these single‐layer electrodes, locally distributed ECoG collection is performed in statuses of resting, penicillin‐induced epileptic seizure, and subsequent suppression with silk‐delivered phenobarbital (Figure 1c). The thin meshed device configuration and soft feature of applied materials enable their conformally compliant attachment onto the ravined cortex surface.^5^ So that high‐quality bioelectrical detection can be achieved to show clear and obviously different spectrum profiles of the above statuses (Figure 1c). The end of the arrays is bonded to the electrode pads with anisotropic conductive film (ACF) to electrically connect to external data acquisition systems (see the “Experimental Section”). Multiple channels of ECoG spectra are collected at detecting point array within the tested area, and are similarly stable in the resting status. The penicillin intraperitoneal injection (2 × 10^6^ units kg^−1^) stimulates epileptiform drastic fluctuations (epileptic spike waves) in the ECoG bioelectrical spectra. The epileptic neural impulsions spatiotemporally spread from the starting focus and propagate in the cortex. The amplitude of fluctuations in ECoG spectra highly depends on the detecting location of electrode channels. The first three ECoG spectra in Figure 1c show intense and sharp fluctuations, indicating the epileptic brain bioelectrical activity spatiotemporally propagating underneath these electrodes. The epileptic spike waves are significantly suppressed after drug (i.e., phenobarbital) delivery via silk matrix.

In Figure 1d, the device on silk is wetting‐induced transforming, conformally mounting onto the rat brain, and simultaneously collecting bioelectrical signals. Colors of signal lattices are associated with the time experienced to provide effective ECoG signals. The dry device is positioned on top of the cortex of a rat (left column of Figure 1d). Only electromagnetic noise but no bioelectrical signal (gray lattices) is detected by the “suspended” electrodes without effective attachment on the brain tissue. After applying normal saline, silk is softened, and the thin electrodes spontaneously and compliantly mount onto the ravined cortex (middle column of Figure 1d). When the silk substrate is completely solved, all 49 channels of the electrode meshes conformally contact the soft and complex biosurface and are able to collect high‐quality ECoG bioelectrical signals (right column of Figure 1d). Additionally, the microstructures imprinted on silk film dissociate along with the conformal mounting and bioelectrical onset of the device (see fabrication details in the “Experimental Section”). The diffractive optical pattern of 532 nm laser beam gradually blurs as shown in Figure 1d. This process is also highly associated with drug delivery via silk matrices and/or packages.^27^ Therefore, these silk‐based diffractive optical elements (DOEs) offer an ideal noninvasive “channel,” parallel to bioelectrical ones, to real‐time monitor their conformal mounting and drug delivery processes. It is of help for the in vivo and in situ operations and multifunctional utilizations of these conformal brain‐integrated electronics.

We investigate the ECoG signal collection via these conformal brain‐integrated electronics, and their configurations are specifically tailored and optimized toward superior bioelectrical detecting performances in vivo (**Figure** **2**). The conformal brain‐integrated electrodes here are suitable for in situ and real‐time monitoring the ECoG signals of the acute epilepsy rat model. Penicillin‐induced epileptiform activities, as a frequently used epileptic model, typically include the enhancement of brainwaves at higher frequencies.^28^ In Figure 2a, the rapid bioelectrical onset associated with the epileptic seizure is clearly detected with a huge change of ECoG frequency (distributed from below ≈5 Hz originally to that up to ≈30 Hz). This transformation of frequency distribution manifests particularly in the power spectral density (PSD) curves in Figure 2b. During penicillin‐induced epilepsy, the bioelectrical energy distributed among ≈0–30 Hz frequencies is much higher than that of resting status (especially those higher frequencies around ≈5–30 Hz). Particularly at 30 Hz, the resting PSD is almost zero, while it reaches ≈5 dB due to the penicillin‐induced epileptic seizure (Figure 2b).

**Figure 2:**
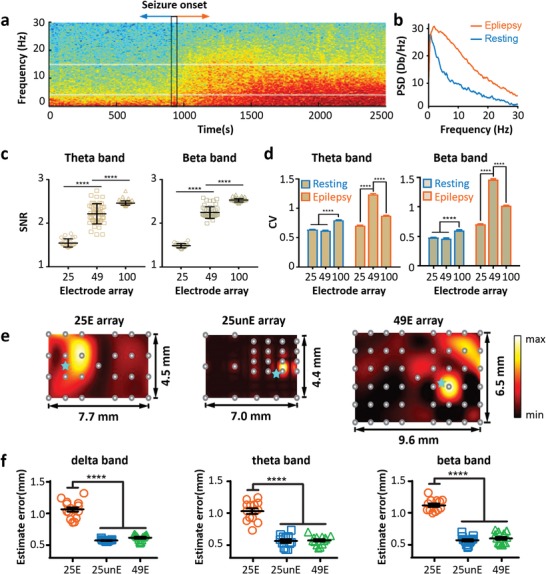
ECoG collections via electrode arrays with different configurations. a) ECoG spectrogram of one channel within uniformly distributed 25 electrode array; the epileptic model in rats is induced by intraperitoneal injection of penicillin sodium. b) Resting and epileptic PSD curves. c) Theta and beta SNR of epileptic rat ECoG with arrays of 24, 49, and 100 channels. d) Theta and beta coefficients of variation (CV) of arrays used in panel (c). e) Local ECoG electrical potential maps in epileptic model rats with customized electrode arrays (left, uniformly distributed 25 channel electrodes; middle, nonuniformly distributed 25 channel electrodes; and right, uniformly distributed 49 channel electrodes). Real epileptic locations are marked with blue stars. f) Estimate errors of electrode arrays used in panel (e) for showing the deviation between ECoG‐revealed and real epileptic locations.

To investigate the influence of device configuration (e.g., channel number, distribution, and electrode gaps) on the collection and subsequent analysis of ECoG data, we compare the electrode arrays with 25, 49, and 100 channels evenly distributed in 1 × 1 cm^2^ areas for ECoG monitoring of different statues (i.e., resting and the penicillin‐induced epileptic cases). For static analysis in theta and beta wavebands, the SNR of collected epileptic ECoG signals is consistently improved along with the increase of channels (left column of Figure 2c).^29^ For evaluating the relationship between the richness of the recorded signals and the number of the electrodes, we calculate the coefficient of variation (CV, a standard statistical measure of a variable's dispersion) across all channels of different electrode arrays. As shown in Figure 2d, the CV of bioelectrical potential signals has the highest value in theta and beta wavebands when the 49 channel array is applied, indicating that the 49 channel array records the neural activity with the highest richness. The data‐collection frequency of the instrument used here is around 1 kHz. If the spacing is too small between electrodes (more electrodes in a specific area), it is difficult to distinguish the epileptic bioelectrical impulses (up to ≈30 Hz) propagating and time dependently occurring at adjacent electrodes. The channel number and/or density higher than an optimal value result in the invalid collection of the spatiotemporally dense neuroimpulses, and consequently, the decreased CV. In the resting status with lower frequencies below ≈5 Hz, the increase of channels from 25 to 49 slightly decreases the CV value of the relatively flat brain bioelectrical spectra. While using 100 channel electrodes, 4 neighboring electrodes collect bioelectrical potential signals around one region. It makes the probability of collecting undistinguished ECoG signals much smaller than the cases of 25 and 49 channels. Therefore, 100 channel arrays have considerably higher CV values in the resting status (Figure 2d). On the other hand, the epileptiform activities are of bioelectrical frequencies much higher than the resting status, as proved in Figure 2a and Figure 2b, respectively. Namely, the ECoG detecting fidelity is directly and inversely proportional to valid data collected and the channel number/density, respectively. The valid ECoG detection approaches to saturation, when the electrode channels and their density increase. Thereby, for operations in this subdural physiological environment of rodent brain, the 49 channel CV is experimentally determined to be ≈100% and 50% higher than the values of 25 and 100 channels, respectively.

Another factor is the matching between the multichannel brain electrodes and the computer‐based data collecting/analyzing system. Micro‐bioelectrode arrays can be facilely fabricated with thousands of channels per 1 cm^2^ by using well‐established lithographical techniques. But the huge amount of data produced during ECoG or other neural detection/monitoring brings about serious burdens of collection and analysis, in terms of processing time and hardware resource, especially impeding in situ and real‐time application cases.^30^ To solve this issue, we further purposely tailor the array configuration in terms of working region, channel number, and especially electrode distribution, specifically for the subdural in vivo ECoG of the penicillin‐induced local epileptic model in rat. In Figure 2e, electrode meshes with 25 and 49 channels, evenly or unevenly distributed, are used to measure the early‐stage ECoG potential maps of penicillin‐induced epileptic seizure in three rats. These arrays are positioned across two hemispheres. In the region with higher channel density of the 25 electrodes unevenly distributed (25unE array) in an ≈7 × 4.5 mm^2^ area (middle column of Figure 2e), the ECoG potential map indicates an epileptic focus area (i.e., the bright yellow area with high bioelectrical potential) to be much smaller than that via an array with 25 electrodes unevenly distributed in a similar area (25E array in the left column of Figure 2e). Also, with the 25unE array, the ECoG map positions the epileptic spot considerably closer to the real focus, namely, more accurate than that with the 25E array. Compared with the 25unE array, the electrodes with 49 channels evenly distribute in the 9.6 × 6.5 mm^2^ area (49E array in the right column of Figure 2e) and provide a bioelectrical mapping with a moderate area determination and similarly precise positioning of the epileptic spot, but a larger monitoring scope. Figure 2f gives the estimate errors (EE) of the electrode arrays used in Figure 2e, quantitatively showing the deviation between the ECoG‐revealed and real epileptic locations (marked with cyan stars). In the delta, theta, and beta bands, 25unE and 49E arrays are of similarly low EE values around 0.5 in comparison with 25E electrodes (about 1.1–1.2).

Improving these BMIs' spatial discernibility, by only optimizing the electrode layouts rather than adding channels, is significant for practices in fundamental research and especially clinical operations. The addition of biointerfacing electrodes results into geometrically increased processing costs of time and hardware, when unscrambling a large volume of raw ECoG signals. For example, up to about 1 TB raw ECoG data can be produced during 24 h monitoring when using a 100 channel device in this work. This obstructs their effective and wide utilization, particularly, in situ/in vivo real‐time applications like clinical cases.^31^ With purposely optimized electrode layouts, a specific region of the rat's cortex surface can be covered and monitored by using these biointerface devices. Namely, enough performances in spatial resolution, accuracy, and monitoring area are flexibly implemented with this layout's customization but no need of excessive addition of channels. These, with lower requirements of accessory elements, data acquisition hardware and processing time help for their operations in space‐limited and complex intracranial environment, and future clinical practical applications.

The neural activities are spatiotemporally dynamic in the brain, as well as their relevant bioelectrical impulses.^9^ For example, the epileptic impulsions spatiotemporally spread from the starting focus toward the whole cortex.^30^ The 25E array in **Figure** **3**a is designed to cover the 7.7 × 4.5 mm^2^ region across the two brain hemispheres of the rat tested. The epileptic bioelectrical activities within the rat cortex are monitored with sufficient spatial accuracy in terms of both resolution and positioning. Thereby, during the seizure and over the cortex across two hemispheres, we can real‐time reveal and monitor the spatiotemporal fluctuations and diffusions of the epileptic impulsions. Penicillin is locally applied onto a spot of the rat cortex after transfrontal craniotomy to induce the epileptic symptoms (see the “Experimental Section” and Figure S6 in the Supporting Information). The time‐dependent sequence of ECoG maps in Figure 3a exhibits that the epileptic neural impulsions start with considerably higher bioelectrical potential at the seizure focus point (Figure 3a‐1). First, they diffuse in the hemisphere in which the seizure focus is present (Figure 3a‐2,a‐3). Then, the impulsions spread into the other hemisphere and induce the increase of the bioelectrical amplitude there. Meanwhile, the ECoG potential in the starting hemisphere goes down to some degree (Figure 3a‐4,a‐5). In Figure 3a‐6,a‐7, partial bioelectrical energy “oscillates” back to increase the potential around the starting spot for the second time. Finally, the excited bioelectrical activities spread all over the two hemispheres (Figure 3a‐8), after ≈30 s since the very first epileptic neural impulsion shows up. With the conformal electro‐biointerfacing devices specifically customized here, the seizure onset is observed to present a spatiotemporal vibration in the cortex, from the starting spot and over the two hemispheres, at its very early stage (within about 30–60 s).

**Figure 3:**
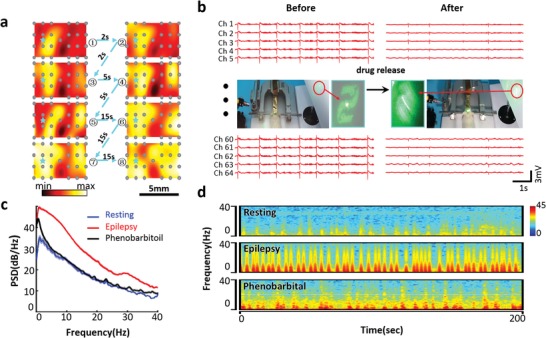
Spatiotemporal diffusion of epileptic ECoG electrical potential and epileptic suppression dynamically monitored with the ultrathin conformal brain‐integrated devices. a) Dynamical diffusion of ECoG electrical potential before an epileptic seizure, monitored with the uniformly distributed 25 electrodes. b) Optical diffraction patterns and ECoG signals changing along with drug delivery via silk matrix. c) PSD curves of resting, epileptic, and phenobarbitoil‐delivered statuses. d) Bioelectrical frequencies of resting, epileptic, and phenobarbital‐treated statuses.

The conformal electro‐biointerface is well‐tailored to reveal these dynamic processes, or even toward the point‐of‐care (POC) capability. The long‐time high‐frequency neural impulses during epileptic seizure usually cause damages to the brain. So, faster release and effect of drugs (e.g., phenobarbital) onto the brain, compared with traditional methods via intravenous injection and blood circulation, are in significant favor of the epilepsy treatments. Silk has versatile capabilities in stabilization and controllable release of medicines, especially, along with superior processability enabling the parallel monitoring of the drug‐delivery processes.^32^ Here, based on the spatiotemporal mapping of epileptic bioelectrical activities in the cortex, we in situ apply phenobarbital onto the brain in epileptic seizures with controllable medicine delivery from silk matrix, and toward the concept of POC therapies (Figure 3b). About 10 mg of phenobarbital loaded in the silk matrix is released, and directly diffuses in cerebrospinal fluid to the brain tissue of the rat in an epileptic seizure. The silk‐delivered phenobarbital considerably stabilizes the turbulent ECoG spectra as shown in Figure 3b. The ECoG frequency and amplitude of the epileptic rat are lowered back to approximately normal status. As phenobarbital is released from the silk matrix to treat epilepsy, the PSD curve goes down back to be similar with that of the resting status (Figure 3c). Also, Figure 3d demonstrates that the high ECoG frequencies during epilepsy are significantly weakened after applying phenobarbital. That is, the epileptic symptoms are effectively suppressed. It is noted that the PSD of epilepsy induced by directly and locally applying penicillin to the brain is up to ≈20 dB Hz^−1^ at 30 Hz (Figure 3c) and much higher than ≈5 Hz via the intraperitoneal injection (Figure 2b). According to this ECoG information, we specifically tune and use a higher dose (i.e., ≈10 mg loaded in the silk film) of phenobarbital.

To parallelly real‐time monitor the drug‐release process, holographic micropatterns are designed and imprinted as DOEs on the silk film. As‐needed therapeutic molecules (i.e., ≈4 mg of phenobarbital here) can be facilely loaded by aqueous blending and well stabilized in the silk pad fabricated with DOEs (see the “Experimental Section” and the Supporting Information). The phenobarbital‐releasing processes are associated with silk dissolution, structural collapse of the DOEs, and the fading and blurring of the diffracted optical pattern (insets of Figure 3b).^11^ The legible optical “readouts” faithfully indicate the drug release and treating effects, parallel to the ECoG spectra. Although proof‐of‐concept is exhibited here, it may be of help for fundamental and/or clinical operations using these multifunctional electro‐biointerface devices, particularly for the cases requiring in situ, real‐time, and POC features (e.g., dynamic ECoG investigating therapeutic effects of new medicine on animal models, POC monitoring, and treating during surgeries).

Neural coding/decoding, as one of the important techniques for the fundamental revelation of cerebral activities and further biomedical diagnosis and treatments, refers to the artificial reconstruction and revealing of stimuli (e.g., sensory) already encoded and represented in the brain.^33^ To our knowledge, a few studies on cracking brain's neural codes are practically investigated previously with the strategy of implantable conformal bioelectronics, although the good potential is the proof of concept exhibited for times.^34^, ^35^ Owing to their biocompatible and conformal direct coupling onto cortex, the well‐tailored soft electrodevices in this work provide the brain‐machine interfacing and ECoG bioelectrical detection with high quality and fidelity. For instance, their EE, SNR, and CV are adjustable and up to ≈0.5 mm, ≈2.5, and ≈1.5, respectively, for specific designs as needed (Figure 2). It endows these silk‐enabled conformal electro‐biointerfaces with applicability to more in‐depth neural analyses like neural decoding. In **Figure** **4**, ECoG signals of five stimulating models are collected and feature‐coded for deducting external stimulations via bioelectrical information of brain activities. Rats are subjected to the idle status, as well as visual (light), current, small and large dose (2 and 10 mg) ketamine stimulations, and 49E electrode arrays are used (see the “Experimental Section” and the Supporting Information for a detailed description). These stimulating models are dynamically and reversibly implemented, and meanwhile monitored with these conformal electro‐biointerfacing devices. Respectively, the 2 mg ketamine‐treated, 10 mg ketamine‐treated, idle, visually (light) stimulated, and current‐stimulated statuses show increasingly anabatic neural activities (e.g., potential amplitudes and frequencies) in the ECoG spectra and maps (Figure 4a).

**Figure 4:**
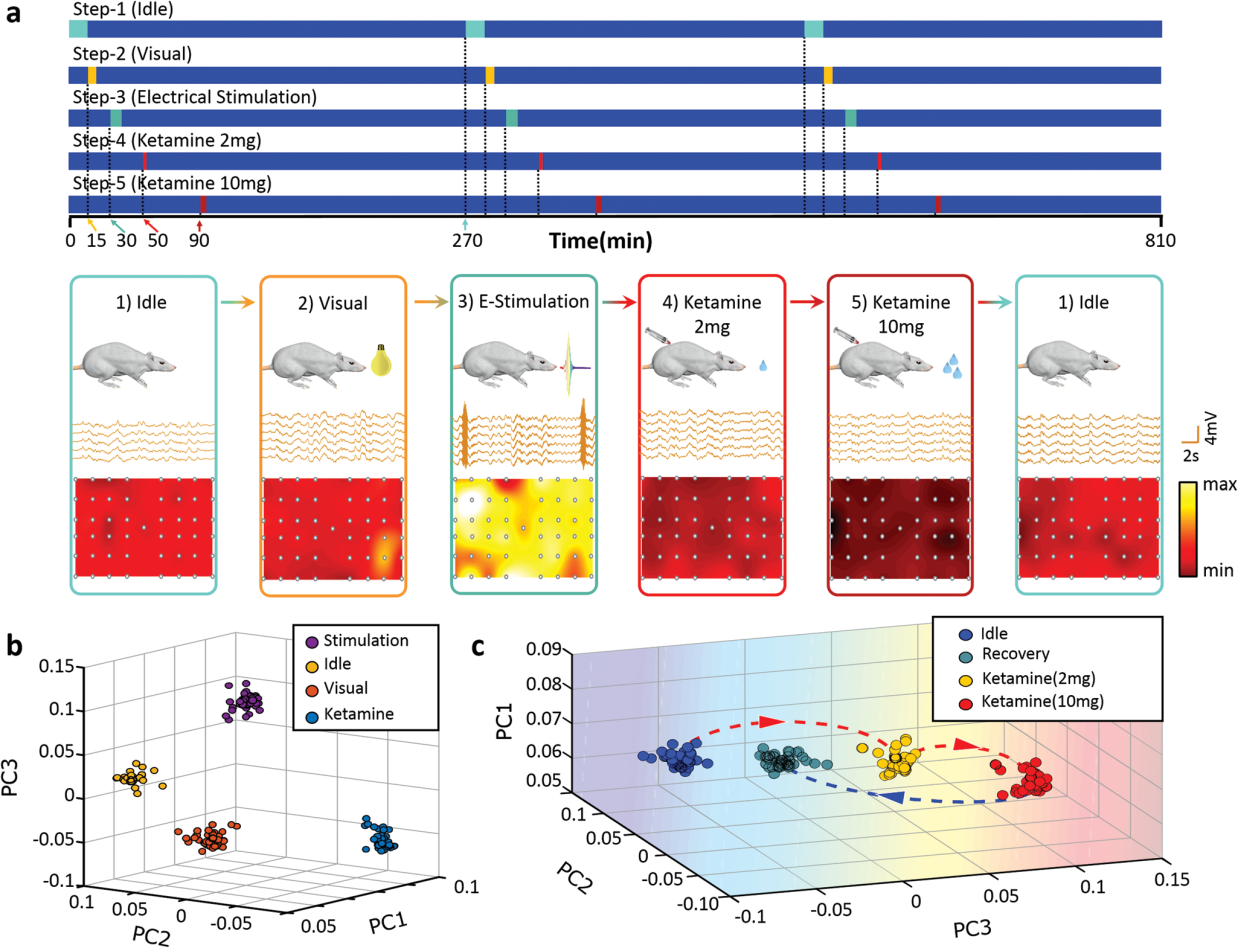
Neural encoding of idle status, visual (light) stimulation, current stimulation, 2 and 10 mg ketamine stimulations, via these conformal biointerfacing devices. a) ECoG ensemble patterns, spectra and 10 s energy statistic maps of statuses (idle, current stimulation, visual stimulation with stroboscopic frequency randomly at 6, 12, 14, 18 Hz, and 2/10 mg ketamine stimulations). The marked spot in the third ECoG map of panel (a) is the current stimulating position. b) Dimension‐reduced results via PCA of statuses in panel (a) (ketamine, 2 mg). c) Chronological PCA results of idle, initial (within 5 min before applying ketamine) and developing (a 5 min period, ≈40 min after applying ketamine) periods of 2 mg ketamine stimulation, and 10 mg ketamine stimulation.

Compared with pathological epileptic cases associated with intense neural impulses, the models for neural decoding here produce ECoG with minor differences. To clearly and intuitively identify the brain bioelectrical activities in correspondence to different statuses/stimuli, the information needs to be further abstracted and analyzed from the ECoG spectra (i.e., shape, amplitude, and frequency) and the ensemble patterns in Figure 4a. PCA is then employed for further verifying and visualizing distinct brain states encoded by the recorded ECoG signals. As an unsupervised, data‐driven approach, PCA transforms the data into linearly uncorrelated components with the same (or less) number of feature dimensions, ordered by the amount of variance explained by each component, which provides an intuitive representation of the recorded dataset (more details in the “Experimental Section” and Supporting Information). Revealed by PCA processing the ECoG data and reducing their dimensionality, brain electrical activities of above models (i.e., current‐stimulated, idle, visual, and 2 mg ketamine‐anesthetized statuses) separably tend to approach respective dimensions in Figure 4b. Furthermore, in Figure 4c, ECoG PCA results distinguish the dynamic processes of idle status, ketamine anesthetization with different doses (i.e., 2 and 10 mg), and the succedent recovery. Along with enhancing the anesthetization, ECoG data distribution after PCA decoding characteristically shifts toward the positive direction of PC3. So that we can infer the anesthetization level by investigating and comparing the data distributions in the PC2 dimension. As a proof‐of‐principle demonstration here in Figure 4c, PCA‐processed data distribution changes backward along the PC3 negative direction to the original idle status, when the rat recovers after applying 10 mg ketamine. It proves the safety of 10 mg ketamine application to the rat. However, the overdose injection of chloral hydrate (i.e., 4 wt%, 5 mL) excessively and irreversibly suppresses brain activities and causes the death of the rat (Figure S9, Supporting Information). This ECoG neural decoding can enable visualized real‐time verifications of pharmacological therapeutic, toxic and side effects, as well as dose setting.

Constructing the purposely soft bioelectronics with well‐tailored biocompatible material and device configuration accommodates their biointerfacing, especially, for neural tissues or organs like brains with kPa‐level moduli and convoluted topography. The soft/flexible feature is indispensably applied in both conformal BMIs here^36^ and injectable neural probes,^37^, ^38^ on the surface and inside the cortex for specific applications, respectively. Notably, properly thinned and softened, namely, ultraflexible, Au micro/nanoelectrodes are in favor of, and already proved to have, superior chronic in vivo biocompatibility without acute negative impacts or damages to the brain.^36^ Moreover, as devices are micro‐ or even nanominiaturized, the soft bioelectronic systems can be implanted (or injected) in the folded state through minimally invasive procedures.^36^, ^37^, ^39^

The surrounding tissues hamper the accurate unfolding and visible positioning of electroprobes delivered in the cortex, which relies more on their spontaneous processes after injecting. However, the conformal BMIs here are customized with array layouts for specific cases, and have inherent convenience in manageably localizing onto targeted detecting spots as needed. This facilitates their practical and clinically applicable utilizations. By taking epilepsy as an example here, the seizure bioelectrically starts from one spot in the brain, and the impulses propagate and finally spread over the entire cortex across two hemispheres. Its ECoG investigations and other cases, for instance, of local brain surgeries (typical brain diseases) require precise and controllable localization, as well as a relatively large monitoring area. Without enormously increasing time and hardware resources, the customization of evenly or unevenly arrayed electrodes enhances ECoG monitoring performances specifically for applications in complex encephalic environments.

These soft and/or flexible BMI strategies, including the conformal cortex‐surface electrodes and injectable in‐cortex electroprobes, are integrated with the ingenious supporting and/or delivering with aqueous or resorbable/degradable solid matrices.^5^, ^7^ In this work, the improved compliantly wrapping‐on contacts spontaneously form during their substrate‐dissolving delivery by using synthetic and bioderived polymers (i.e., silk), and lead to high‐quality bioelectrical probing on the cortex surface. Silk owns special merits in designing dissolving/degrading periods as required, by easily tailoring the size, conformation, crystallization, and crosslinking of the protein molecules with flexible chemical (e.g., covalent crosslinking and modification), physical (e.g., heating and shearing), and biological (i.e., genetic engineering) methods.^5^, ^11^, ^40^ Importantly, silk not only supplies reconfigurable support and delivery for the “conformal” feature of these soft BMIs, but also enables the integration of multiple functions toward “all‐in‐one” concept. Silk is known as an ideal optical biopolymer,^11^ and loading/releasing matrix and/or stabilizer of bio‐specimens and medicines (e.g., phenobarbital here, penicillin, and vaccines).^31^, ^41^ It possesses diversiform and facile processabilities such as imprinting, inkjet printing, electron‐beam lithography, and photolithography.^11^, ^42^ Thanks to these properties, this work also in principle demonstrates the organic combination of functions (e.g., optical visualization of compliant mounting and drug releasing, and in situ drug delivery), supplementary and helpful to space‐saving high integration and multipurpose clinical POC.

Previous investigations of soft conformal biointerfaces mainly perform ECoG signal collections for ability demonstration.^5^, ^16^, ^35^, ^43^ In this paper, we compared arrays with different electrode densities, and customized the optimal 49 channel for ECoG specifically in the rodent model. Also, unevenly distributed ECoG arrays showed superior performances (e.g., monitoring area and spatiotemporal resolution) with fewer channels have been demonstrated as well. Besides, by specifically designing toward practical neural applications, we have accommodated these silk‐based conformal BMIs and their ECoG data analysis toward POC conceptual real‐time and in situ spatiotemporal brain monitoring. A specific demonstration with the epilepsy model in rats is implemented to identify and observe the spatiotemporal vibrating spread of epileptic seizures in the cortex, and, “at that time,” suppress the symptoms via in situ phenobarbital delivery from silk. Furthermore, these conformal biointerfacing electrodevices are used to code/decode neural and/or brain activities of rats in a series of statuses. Accordingly, an ECoG PCA investigation is conducted to evaluate pharmacological effects (i.e., dosing, anesthetizing effects, and recovery) of medicines, such as ketamine and chloral hydrate. Especially, we tune the doses of ketamine, and, by PCA decoding, distinguish the imperceptible changes of neural activities which are difficult to be directly mentioned by ECoG spectra. This method is of practical potential to visualize the degree of anesthesia and further monitor the recovery or change of anesthesia states. Silk‐centered engineering can lead to integrating more functions as required by the practical research and clinical usages. Based on these investigations, silk‐based conformal electro‐biointerfaces can be exploited for more diverse and in‐depth electroneurographic utilizations in more kinds of animal models. Novel opportunities may be opened to improve performances (e.g., effects, chronic biocompatibility, and/or customizable transience) of relevant diagnosis and therapies, or to promote frontier discoveries in neural/brain scientific researches especially via their upgraded characteristics.

## Experimental Section

*Silk Preparation*: *Bombyx mori* silk fibroin was prepared with the established protocols. *Bombyx mori* cocoons were boiled in 0.02 m Na_2_CO_3_ (Sigma‐Aldrich, USA) aqueous solution for 30 min, and then rinsed with distilled water for three times (30 min per time) to remove the Na_2_CO_3_ and sericin. After drying in air for ≈12 h, the degummed cocoons were dissolved in 9.3 m LiBr (Sigma‐Aldrich, USA) solution at 60 °C for ≈4 h. This solution was dialyzed for 2 days in distilled water by using Slide‐A‐Lyzer dialysis cassettes (molecular weight cutoff, MWCO = 3500, Pierce, USA). Subsequently, the solution was centrifuged two times for 20 min at 18000 rpm. The final silk concentration was determined to be about 6–7 wt% by measuring the volume of the solution and the final dried weight.

*Device Design and Fabrication—Fabrication of Single‐Layer Device*: The wet oxidation process was used to create a 2 µm thick silicon oxide as the sacrifice layer on a silicon wafer. Then, it was spin‐coated with a 2 µm thick layer of PI. After patterning a 3 µm layer of AZ‐100 photoresist on PI by photolithography, Cr/Au patterns (150/1500 Å) were electron‐beam evaporated. Another 2 µm thick PI layer was spin‐casted on the metal layer, followed by the sputtering process of aluminum (Al, 3000 Å). The photoresist (AZ‐100) mask pattern was photolithographically made on Al to etch out Al patterns, in the acid corrosive liquid (a mixture of hydrochloric and acetic acid). With these Al patterns as the mask, PI layers were then etched into patterns with microwave plasma. The meshed structures and exposed pads were made by applying two different masks and etching depths. The soft electrodes were separated from the wafer after etching off the left Al with acid corrosive liquid and the SiO_2_ sacrificial layer with buffered oxide etch (BOE) containing hydrofluoric acid and ammonium fluoride). These soft electrodes were transferred onto dry silk films, and rinsed with acetone to enable firm attachments. Finally, they were thermally pressed (170 °C and 0.6 MPa) to connect the soft electrodes with a flexible printed circuit (FPC) by using ACF cable.

*Device Design and Fabrication—Fabrication of Multilayer Device*: Step 3 in Figure S3 (Supporting Information) was repeated to integrate one or more electrode layers with various functions. For instance, the electrodes used in Figure S6 (Supporting Information) were integrated with electrical‐stimulating probes. Refer to the Supporting Information for more detailed descriptions on single‐layer and multilayer devices.

*Epilepsy Model*: Intraperitoneal penicillin injection in a Sprague–Dawley rat induced the model of multifocal epilepsy. Cortical penicillin injection induced the epileptic model with the single focus at injecting spot (see Figure S6 in the Supporting Information).

*ECoG Animal Experiments and Ethic Documents*: Experiments were conducted in accordance with the ethical guidelines and with the approval of the Huashan Hospital. ECoG recording was operated with the Blackrock Cerebus system. The sampling frequency of original date was 10 kHz and transcoded to MAT formats at 1 kHz.

*ECoG Data Analysis*: Spectrogram analysis (Figure 2a) captured the frequency content of the recorded ECoG signal. The spectrogram of an ECoG signal was calculated from 0 to 30 Hz during the time period of 2500 s, the number of frequency values was 1024 within 0–30 Hz, and the time bin size was 1 s. There were three states occurred within this 2500 s time window, that is, the resting state, seizure onset, and the epilepsy state. The horizontal gray lines at 4 and 15 Hz denote the separations of delta band, theta bands, and beta band.

The PSDs of recorded ECoG signal (Figures 2b and 3c) were calculated in three frequency bands, i.e., delta band (1–4 Hz), theta band (4–8 Hz), and beta band (13–30 Hz). Before filtering the recorded signals to these three frequency bands, a 1 Hz high‐pass filter (fourth‐order Butterworth, cutoff frequency 1 Hz) was applied on each recorded channel. The bandpass filters were then constructed by using Matlab function “design(fdesign.bandpass(‘N,F3dB1, F3dB2', 10, *a*, *b*, 1000), ‘butter'),” where *a* and *b* are the cutoff frequencies for each frequency band. The fast Fourier transform was applied to the recorded ECoG signal, and the resulting frequency resolution was 0.05 Hz.

In the SNR analysis (Figure 2c), the noise is defined as the interquartile range (IQR) as a measure of the variability (noise) across all recorded signals by using different electrode setting (25, 49, and 100 electrodes). SNRs were then calculated as the averaged (median) spectral power values divided by the noise level in each frequency band.^35^

In the present analysis, CV is defined as the ratio of the standard deviation σ to the mean *µ* of the amplitudes of all the channels in the electrode array. CVs were calculated for each recorded time point during the experiment; the statistics shown in Figure 2d is mean ± standard error (SE) of the CVs.

Topographic maps of signal's amplitude in Figures 2e, 3a, and 4a were generated by using a two‐step analysis. In Step 1, the signal of each electrode was *Z*‐scored for eliminating the variabilities of different electrodes, and the signal's amplitude for each electrode was then calculated with a 0.1 s time window. In Step 2, the amplitude of each electrode was arranged according to its physical location, and the topographic maps of the signal's amplitude were visualized as interpolated maps in a 0.01 mm spatial resolution.

The global maximum point was assigned within the first 10 s after the injection as the estimate onset site of the epileptic seizure, and the estimate error was then calculated as the spatial distance between the estimate onset site and the actual injection position, as shown in Figure 2f.

PCA was applied for demonstrating the approachability of the proposed electrode array in the brain decoding project. The PSDs of the recorded ECoG signal in all three frequency bands (delta band, theta band, and beta band; the PSDs of these bands are rearranged as a 1D vector) were served as the input of the PCA method. The results of PCA analysis were visualized in a 3D space based on the first three principal components, as shown in Figure 4b,c.

*Drug Loading/Releasing via Silk Matrix*: Needed medicines (i.e., penicillin and phenobarbital) were blended into silk proton solution and the aqueous hybrids were casted into easy‐to‐use films and/or pads. The dosage of medicines was adjusted by controlling the amount of the drugs mixed/loaded, according to specific cases.

*DOE Design and Fabrication*: To generate diffractive diffusers, numerical simulations were performed by the commercial software, namely, LightTrans VirtualLab 5. Diffractive diffusers were decided and the scattering of light was produced into an arbitrary 2D light pattern. This light‐intensity pattern was conversed into GDSII format and used as the mask to fabricate DOEs. The working wavelength of the input beam was set at 450 nm, and the optical setup was selected as paraxial far‐field. The construction of silk DOEs was introduced in the previous work, which described more details.^11^ Briefly, a self‐assembled monolayer of octadecyltrichlorosilane was deposited on the Si‐mask surface by molecular vapor deposition for the hydrophobic treatment. Then, silk proton solution was casted into the structurized Si mask, and the silk films/pads with DOE configurations could be peeled off the mask after 24 h drying in the ventilator.

*Fabrication of Silicon Masks*: SiO_2_ was thermally grown with a thickness of 600 nm on the silicon wafer. A spin‐coated layer of AZ‐100 photoresist was photolithographically patterned as the mask for the following reactive ion beam etching of exposed SiO_2_. After photolithography, the residual photoresist was removed by microwave plasma treating by a wet etching process using H_2_SO_4_.

*Animal Experiments (Silk‐DOE Degradation)*: Rats (6–8 week old) were anesthetized with an intraperitoneal injection of chloral hydrate (4 wt%, ≈2 mL). After the craniotomy, electrode devices were attached to the cortex surface for the real‐time ECoG monitoring (more details are provided in the Supporting Information). According to the epileptic symptoms, silk films with proper dosages of drugs are directly positioned on the surface of the cortex (e.g., an ≈0.6 g silk pad containing ≈0.7 wt% phenobarbital was used here for effectively suppressing the ≈10 time min^−1^ seizures). After dropping the saline solution, the loaded therapeutic molecules were released gradually along with the dissolution of the silk matrix. A 532 nm laser beam was projected onto the silk DOEs with an incident angle of around 10°–20°, and the clear optical pattern appeared after reflecting. The holographic optical pattern changes from clear to blurred during the silk dissolution and drug release.

*Statuses for Neural Decoding*: 1) Idle: the rat was tested in quite a dark environment. 2) Visual: the rat was stimulated with a white light‐emitting diode (LED) with ≈7500 lux illuminance. Ten times of stimulation (the 40 s resting after 10 s illumination per time) were performed after which the rat was in the resting state. 3) Electrical stimulation (E‐stimulation): ten times of current stimulations (the 30 s resting after 1 mA current stimulation for 2 s per time) were applied through the stimulating electrode integrated into the conformal BMIs at the marked spot in the third ECoG map of Figure 2a, after which the rat was in the resting state. 4) 2 mg ketamine: ECoG of the rat was recorded for 30 min after 2 mg ketamine intraperitoneal injection. 5) 10 mg ketamine: ECoG of the rat was recorded for 3 h after 10 mg ketamine intraperitoneal injection.

## Conflict of Interest

The authors declare no conflict of interest.

## Supporting information

Supplementary
