# Supplementary material for: Silk‐Enabled Conformal Multifunctional Bioelectronics for Investigation of Spatiotemporal Epileptiform Activities and Multimodal Neural Encoding/Decoding
Source: Adv Sci (Weinh). 2019 Mar 7;6(9):1801617. doi: 10.1002/advs.201801617 (PMC6498121; doi:10.1002/advs.201801617)
Supplement: Supplementary file 1 — Supplementary [file ADVS-6-1801617-s001.pdf]

Copyright WILEY-VCH Verlag GmbH & Co. KGaA, 69469 Weinheim, Germany, 2019.

ADVANCED SCIENCE Open Access

Supporting Information

for *Adv. Sci.,* DOI: 10.1002/advs.201801617

Silk-Enabled Conformal Multifunctional Bioelectronics for Investigation of Spatiotemporal Epileptiform Activities and Multimodal Neural Encoding/Decoding

Zhifeng Shi, Faming Zheng, Zhitao Zhou, Meng Li, Zhen Fan, Huanpeng Ye, Shan Zhang, Ting Xiao, Liang Chen, Tiger H. Tao, Yun-Lu Sun,* and Ying Mao**

Copyright WILEY-VCH Verlag GmbH & Co. KGaA, 69469 Weinheim, Germany, 2019.

Supporting Information

Silk-enabled Conformal Multifunctional Bioelectronics for Investigation of Spatio-temporal Epileptiform Activities and Multi-modal Neural Encoding/Decoding

Faming Zheng, Zhifeng Shi, Zhitao Zhou, Meng Li, Zhen Fan, Huanpeng Ye, Shan Zhang, Ting Xiao, Liang Chen, Tiger H. Tao*, Yun-Lu Sun*, Ying Mao*

Faming Zheng, Dr. Zhitao Zhou, Shan Zhang, Ting Xiao, Prof. Tiger H. Tao
State Key Laboratory of Transducer Technology, Shanghai Institute of Microsystem and Information Technology, Chinese Academy of Sciences, Shanghai, 200050, China
E-mail: tiger@mail.sim.ac.cn

Dr. Zhifeng Shi, Dr. Zhen Fan, Prof. Liang Chen, Prof. Ying Mao
Department of Neurosurgery, Huashan Hospital of Fudan University, Wulumuqi Zhong Road 12, Shanghai, 200040, China
E-mail: maoying@fudan.edu.cn

Dr. Meng Li
The Rowland Institute, Harvard University, 100 Edwin Land Blvd, Cambridge, MA 02142, USA

Huanpeng Ye
State Key Laboratory of Mechanical System and Vibration, Shanghai Jiao Tong University, Shanghai 200240, China

Shan Zhang, Prof. Tiger H. Tao
School of Graduate Study, University of Chinese Academy of Sciences, Beijing 100049, China

Ting Xiao
The Key Laboratory of Resource Chemistry of Ministry of Education, Shanghai Normal University, Shanghai, 200234, China.

Prof. Tiger H. Tao
School of Physical Science and Technology, Shanghai Tech University, Shanghai 200031, China

Dr. Yun-Lu Sun
State Key Laboratory of Integrated Optoelectronics, College of Electronic Science and Engineering, Jilin University, 2699 Qianjin Street, Changchun 130012, China.
E-mail: sunyunlu@jlu.edu.cn

Keywords: Brain-machine interfaces, Conformal transient bioelectronics, Silk, Electrocorticogram, Neural Decoding

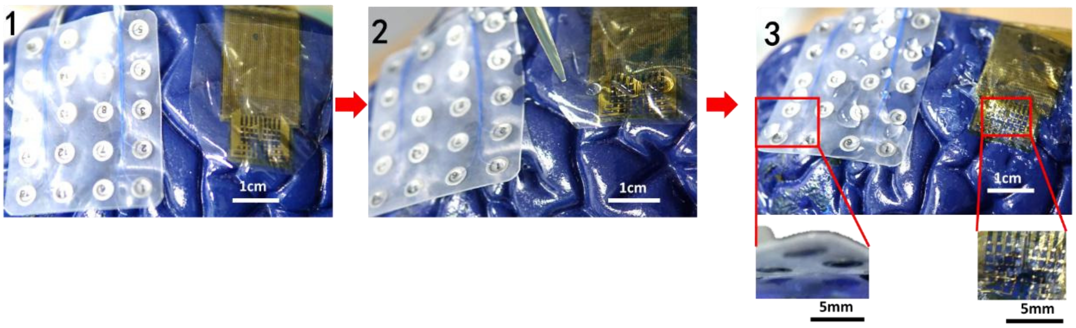

Figure S1. Cortex attachments of silica-gel-supported ECoG electrodes frequently used in present clinical applications and soft BMIs in this work. Soft BMIs in this work show higher probe density, stable attachments on ravined cortex surface, and thereby remarkably improved brain-computer interfacing with their conformal contacts.

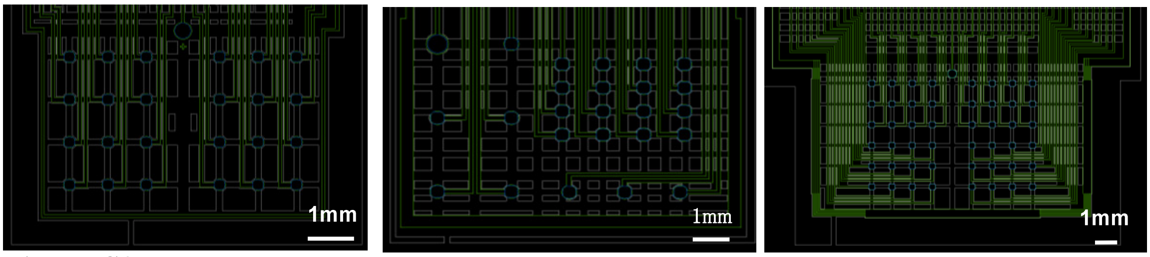

Figure S2. Configuration design of single-layer electrode arrays.
(24-even-electrode,24-uneven-electrod, 49-even-electrode)

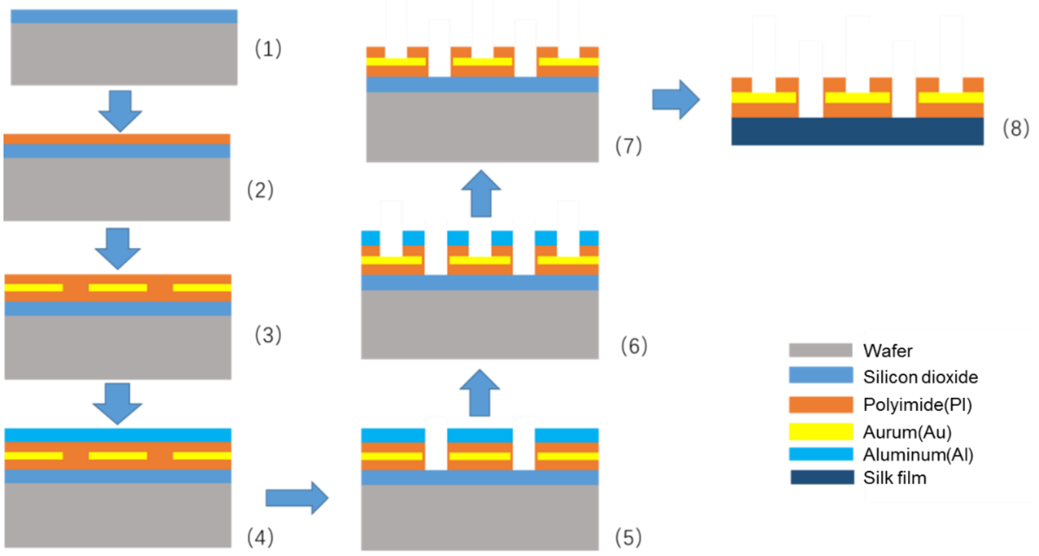

Figure S3. Schematics of fabrication procedures of silk-supported BMI electrodes (one layer for demonstration).

The procedures include:

1. a 2-µm-thick SiO_2 sacrificial layer is made on cleaned a 4-inch Si wafer by LPCVD.
2. a 2-µm-thick polyimide (PI) film (supporting layer) is spin-coated and thermally cured on the SiO_2 surface.
3. Au patterns (150nm thick) with an adherent Cr layer (15nm thick) are fabricated on the PI layer by thermal evaporation, after which a second 2-µm-thick layer (insulating layer) of PI is made on the Au/Cr layer.
4. A layer of Aluminum (Al) is sputtered on the insulating PI layer.
5. The Al layer is patterned as the mask and the insulating PI layer is patterned with meshed structures of the designed electrode array by plasma-etching.

6. For the second time, the Al layer the top PI layer are patterned sequentially to expose the Au electrodes.
7. Remove residual Al.
8. Remove the SiO_2 sacrificial layer by immersing in BOE aqueous solution (Buffered Oxide Etch, 49% HF:40%NH_4F=6:1, all by volume).

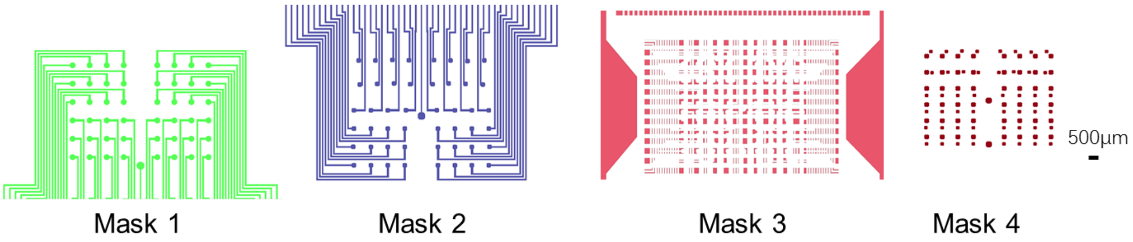

Figure S4. Configuration design of multi-layer electrode arrays. Multiple devices (e.g., bioelectrical probes and current-stimulating electrodes) can be integrated into these multi-layered BMIs for multi-functions.

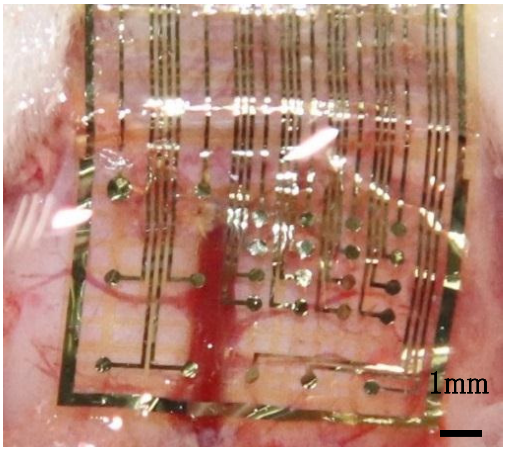

Figure S5. An electrode array with unevenly distributed 25 channels.

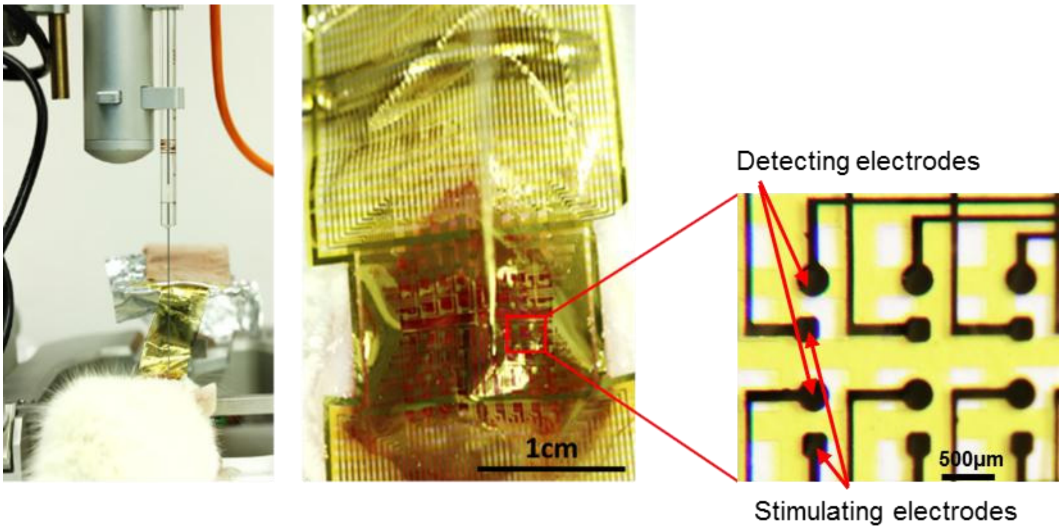

Figure S6. Left: direct subcranial penicillin injection (2.5-μL 800-unit/μL penicillin sodium aqueous solution) to locally induce epilepsy in a rat.
The depth of needling microsyringe was 1mm, then return needled 10 mm. Right: conformal BMI array integrated with bioelectrical probes and current-stimulating electrodes.

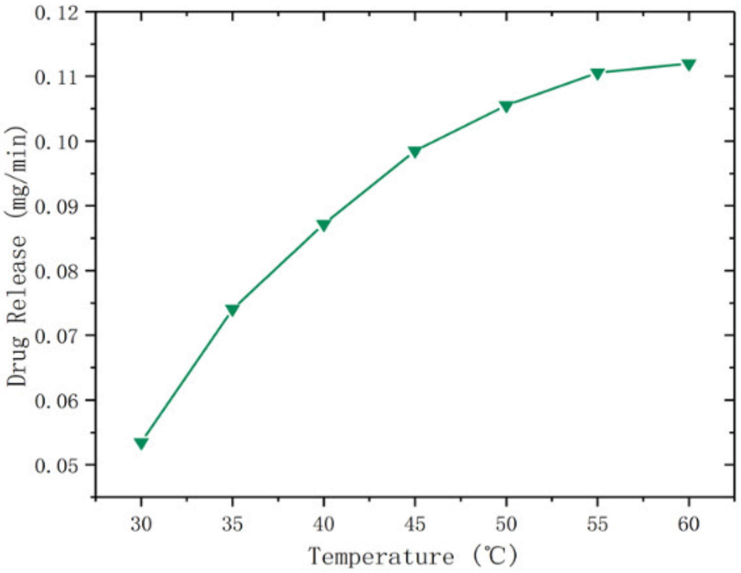

Figure S7. Drug-releasing rates from silk matrix under different temperatures (penicillenic acid with good stability and an absorption peak at 324nm).

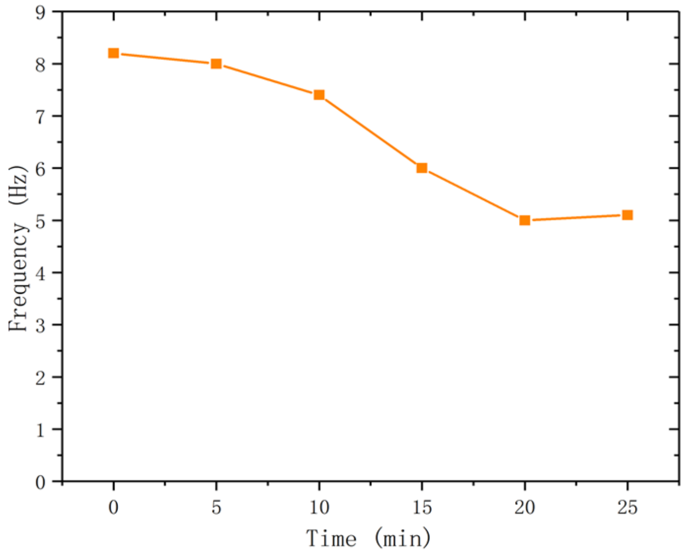

Figure S8. Time-dependent ECoG frequency change after in-situ delivering 10-mg phenobarbital from silk matrix for suppressing the epileptic seizure.

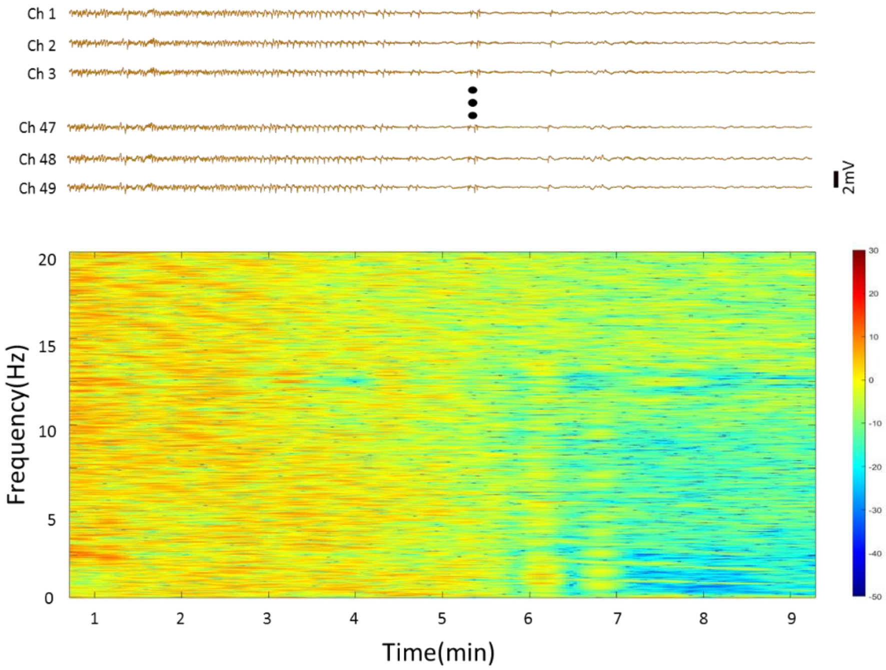

Figure S9. ECoG signals fading away after applying over-dose chloral hydrate. Over-dose chloral hydrate (i.e., 5mL 4wt% aqueous solution for a rat) can irreversibly suppress neural activities and be lethal.

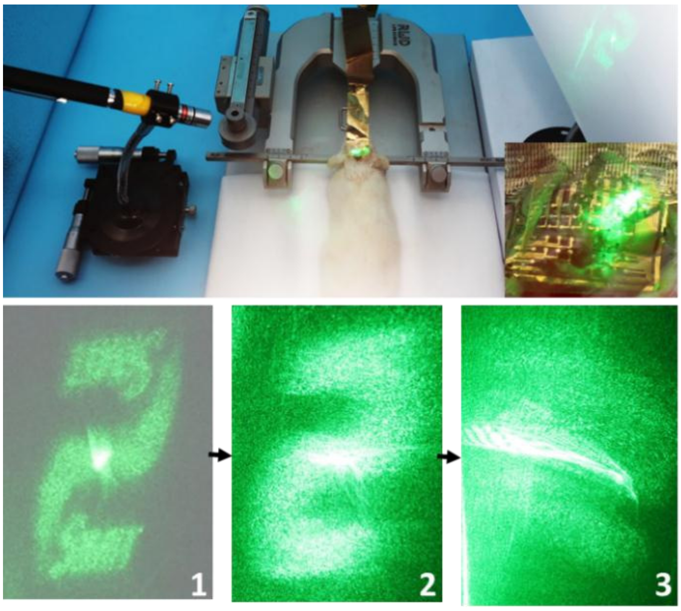

Figure S10. Optically monitored drug release from the silk matrix. The holographic pattern changes from clear to blurred during the silk dissolution and drug release.

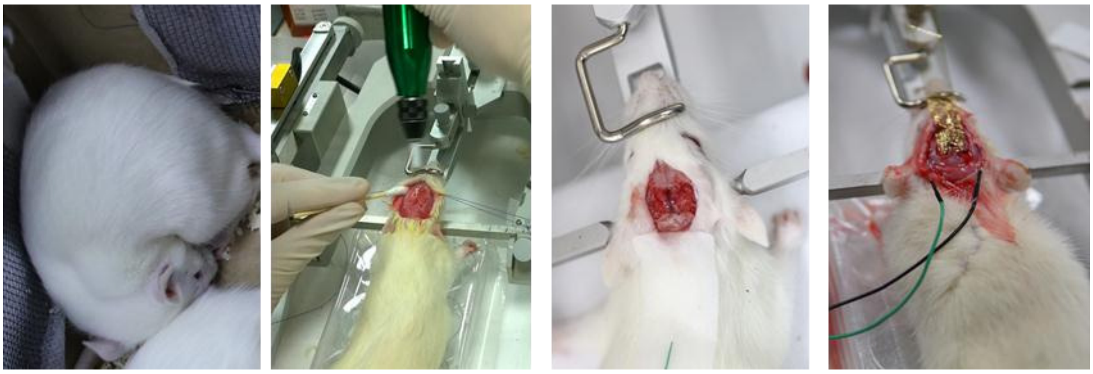

Figure S11. Craniotomy of a rat. The rat is fixed on the animal testing stand after anesthetization. A $1 \times 1 cm^2$ window is opened through the cranium and meninges to expose the brain. The wetting, dissolving, and wrapping processes of silk-supported conformal BMIs are as described in the previous work in the literature to compliantly mount the devices on the ravined cortex surface. [5]
